# Absence of *Brucella canis* Detection in Dogs from Central Italy: Implications for Regional Surveillance and Zoonotic Risk

**DOI:** 10.3390/epidemiologia6040071

**Published:** 2025-11-03

**Authors:** Maria Luisa Marenzoni, Sabrina Attura, Brigitta Favi, Maria Teresa Antognoni, Maria Beatrice Conti, Andrea Felici, Carmen Maresca, Eleonora Scoccia, Maria Rita Bonci, Alessia Pistolesi, Simona Zanghì, Anna Confaloni, Lakamy Sylla, Daniele Marini, Fabrizio De Massis, Flavio Sacchini, Manuela Tittarelli

**Affiliations:** 1 Department of Veterinary Medicine, University of Perugia, 06124 Perugia, Italymaria.conti@unipg.it (M.B.C.);; 2 Servizio di Sanità Animale, USL UMBRIA 1, 06127 Perugia, Italy; 3 Istituto Zooprofilattico Sperimentale Umbria e Marche “Togo Rosati”, 06124 Perugia, Italye.scoccia@izsum.it (E.S.); 4 Independent Researcher, 06063 Perugia, Italy; 5 Istituto Zooprofilattico Sperimentale dell’Abruzzo e del Molise “G. Caporale”, National Reference Center for Brucellosis, 64100 Teramo, Italym.tittarelli@izs.it (M.T.)

**Keywords:** *Brucella canis*, brucellosis, zoonosis, dogs, public health

## Abstract

**Background**: *Brucella canis* is a zoonotic pathogen associated with reproductive disorders in dogs and represents an emerging public health concern. Dogs are the only known source of infection for humans, and transmission is often associated with close contact, particularly in occupational settings. Reports of canine and human infections in Europe are increasing, underscoring the need for integrated surveillance to assess the risk of introduction and spread. **Objectives**: This study aimed to investigate the possible circulation of *B. canis* in different subgroups of dogs from Central Italy, representing diverse risk contexts (stray, breeding, blood donor, refugee-associated, and previously outbreak-linked dogs), and to generate sentinel data to inform further risk-based surveillance and zoonotic risk assessment. **Methods**: A comprehensive serological, molecular, and bacteriological survey was conducted on 128 dogs sampled in the Umbria region, covering animals from different backgrounds and risk contexts. Blood samples were tested using bacterial culture, real-time PCR, serum agglutination test, complement fixation test, and/or indirect immunofluorescence antibody test. **Results**: All tested dogs were negative for *B. canis*. The upper 95% confidence limit for prevalence was 3.5%, suggesting that widespread circulation is unlikely, although a low/moderate prevalence in specific groups cannot be excluded. **Conclusions**: Although no cases of *B. canis* were detected, the results provide sentinel information and highlight the need for continued risk-based surveillance, particularly in low-prevalence areas to prevent introduction of the infection and to enable early detection in case of occurrence. As dogs are the only known source of human infection, veterinary monitoring plays a pivotal role in mitigating zoonotic risks and supporting One Health strategies for evidence-based control.

## 1. Introduction

*Brucella canis*, an emerging zoonotic pathogen, has been increasingly reported across Europe in humans and dogs, particularly in association with international dog movements [1,2,3,4,5,6]. This pathogen is a significant cause of reproductive failures in dogs and poses considerable public health risks due to its zoonotic potential [5,6,7,8]. Although human brucellosis due to *B. canis* is likely underdiagnosed, given its nonspecific clinical manifestations and the limited use of specific diagnostic tools, cases have been reported in both Europe and the Americas, especially in individuals with close or occupational contact with infected dogs [3,5,8,9,10,11,12,13,14]. In Latin America, particularly in Brazil and Argentina, human infections are more frequently reported, reflecting both higher canine prevalence and greater diagnostic awareness in some areas [6,8,11,12,13].

In addition, worldwide, the growing globalization of pet movements, driven by diverse factors such as adoption campaigns, commercial breeding, trade of purebred breeding dogs, and humanitarian crises, has facilitated the transboundary spread of *B. canis* [2,4,11,14,15,16,17,18]. In particular, the relocation of dogs accompanying refugees from conflict-affected regions has introduced additional biosecurity challenges, as these animals may originate from areas where *B. canis* is endemic. Countries like the United Kingdom, which have seen a rise in cases linked to imported dogs, have emphasized the importance of stricter import controls and risk assessments to identify high-risk countries and prevent the introduction of infected animals [17,19].

On the other hand, *B. canis* is considered also relevant in blood donor dogs, as the pathogen is recognized as transmissible through blood transfusions from asymptomatic animals; therefore, donor dogs represent a category to be monitored [20].

In this context, it is important to characterize the epidemiological situation at the national and regional level. In Italy, systematic data are limited, and some regions may be particularly exposed to *B. canis* introduction and spread due to specific risk factors. Among these, Umbria region represents an example because the region has received dogs from Eastern Europe in connection with refugee flows, hosts shelters with relatively high animal turnover, and is characterized by frequent international movements of hunting and breeding dogs. Hunting dogs in particular, often involved in travels abroad, represent also a substantial proportion of the regional canine population [21], with many of them also serving as blood donors [22]. Moreover, the recent outbreak of *B. canis* in Italy [14], which involved a kennel hosting approximately 600 dogs and represents one of the largest outbreaks reported in Europe, had epidemiological links with Umbria, as dogs originating from the affected kennel were sold into the region before the outbreak was officially recognized.

These conditions further emphasize the vulnerability of Umbria and the importance of targeted surveillance, as surveillance data are essential to inform both veterinary and public health risk assessments and to guide preventive strategies, particularly in regions with limited baseline information. This study aimed to investigate the possible circulation of *B. canis* in dogs from the Umbria region (Central Italy), generating sentinel information to support further risk-based surveillance, early detection, and zoonotic risk assessment within a One Health framework.

## 2. Materials and Methods

### 2.1. Study Design

A cross-sectional study was conducted between July 2023 and February 2024 in the Umbria region, involving collaboration among the Department of Veterinary Medicine of the University of Perugia, public veterinary services, the Istituto Zooprofilattico Sperimentale Umbria e Marche, the National Reference Centre for Brucellosis (Istituto Zooprofilattico Sperimentale of Teramo), and local veterinary practitioners.

To detect a minimum expected prevalence of approximately 5%, with a 95% confidence level and a precision of ±10%, under the assumption of a binomial distribution, the minimum sample size was set at 100 dogs [23]. A total of 128 dogs were ultimately included to account for potential exclusions or unviable samples. However, while the sample size was initially estimated assuming a homogeneous population, the final design involved five subgroups with different risk profiles (stray host in kennels, breeding, blood donor, refugee-associated, and previously outbreak-linked dogs). Per-stratum sample sizes were not sufficient to provide precise prevalence estimates within each subgroup, and the results have to be interpreted as exploratory. In this study, sentinel data are defined as information from risk groups with higher likelihood of natural exposure, used to provide early epidemiological warning of pathogen introduction or circulation, rather than prevalence in the general population [23]. Consequently, the sample size in each group did not result from a pre-planned experimental design, except for the initial overall number, but rather from an opportunistic use of available cases (e.g., dogs undergoing routine veterinary procedures, surveillance investigations, or those accessible through shelters and blood donor programs). Dogs were enrolled during their admission to kennels, breeding facilities, or blood donor programs, until the target sample size was reached. Dogs were sampled from different sources across the province, including three municipal kennels and multiple private facilities, in order to ensure representation of a wide part of the territory and to reflect the diversity of the regional canine population.

Prevalence data are presented descriptively with exact 95% confidence intervals; for groups with zero positives, the upper 95% confidence limit was calculated using the “rule of three” approximation (≈3/n) [24].

### 2.2. Ethical Considerations

Samples were collected during routine veterinary procedures (i.e., standard health checks), requiring no specific ethical approval. Blood samples from donor dogs were anonymized and retrospectively analyzed. For the group of dogs previously associated with a previous *B. canis* outbreak, samples were collected during routine sanitary visits for surveillance purposes, so formal ethical approval was not required. Owners provided informed consent for the use of clinical samples for epidemiological investigation.

### 2.3. Study Population and Sampling

All tested dogs were clinically healthy at the time of sampling, and none was included on the basis of clinical suspicion of brucellosis. Dogs were divided into five groups according to their potential epidemiological role and distinct exposure pathways: (i) shelter dogs, often with high turnover and uncertain health history; (ii) breeding dogs, which may contribute to vertical and venereal transmission and are often moved nationally and internationally; (iii) blood donor dogs, which may represent a risk for transfusional transmission; (iv) dogs accompanying refugees from Ukraine with unknown *B. canis* health status, (v) dogs previously associated with a previous *B. canis* outbreak in Italy and currently under voluntary monitoring by their owners under the supervision of public veterinarians. Hunting dogs were mainly represented within the donor, breeding, and shelter groups.

Samples were stored and transported at 4 ± 2 °C to the National Reference Centre for Brucellosis for testing.

### 2.4. Diagnostic Methods

Based on sample availability and quality, a comprehensive diagnostic approach was applied whenever possible, combining direct and indirect methods to ensure high sensitivity and specificity in detecting *B. canis* at different stages of infection. The approach followed the protocols of the National Reference Centre for Brucellosis [25]. Direct (blood culture and real-time PCR ) and indirect tests (Serum Agglutination Test [SAT], Complement Fixation Test [CFT], and/or Indirect Fluorescent Antibody Test [IFAT]) were applied in parallel, so that a dog was considered positive if at least one assay yielded a positive result.

The two diagnostic arms are complementary, targeting different phases of infection—direct tests detect early bacteremia, while serological tests identify post-seroconversion stages. Culture is highly specific and has good sensitivity during the peak bacteremic phase (5–30 weeks post-infection), but its performance declines thereafter, as bacterial presence in the blood becomes intermittent [14,25]. PCR shows >90% sensitivity and near-perfect specificity, while serological tests detect antibodies from 5–8 weeks post-infection, with a combined sensitivity of ~98–99% and specificity of ~78–90%. Because the diagnostic windows are not perfectly overlapping, their combination is advantageous, as it maximizes overall case detection across stages of infection [14,25]. This strategy is particularly valuable for surveillance in low-prevalence regions, where subclinical cases may otherwise be missed [25,26].

Whenever feasible, blood samples underwent both direct (bacteriological culture, real-time PCR) and indirect (serological) testing. The diagnostic test panel applied to each group varied according to sample availability and quality, reflecting contingent constraints related to sampling. For blood donor dogs, only serological tests were performed using previously stored plasma, as no whole blood was available for direct testing. For dogs accompanying refugees, direct tests were conducted in all cases, while serological testing was feasible in only eight samples by SAT and CFT; in the remaining dogs, only IFAT could be applied, as most sera were unsuitable for SAT and CFT due to hemolysis (Table 1).

Briefly, blood cultures were performed using Farrell’s selective agar and enrichment broth, incubated at 37 ± 1 °C for up to one month, with weekly subcultures. Colonies indicative of *Brucella* spp. were subsequently identified via PCR.

Real-time PCR was performed not only on culture-positive samples but also directly on DNA extracted from whole blood, in order to maximize the likelihood of detecting bacteremia, which may occur as early as two weeks post-infection. Real-time PCR was carried out using the Genesig™ Advanced Kit (Genesig, York House, School Lane, Chandler’s Ford, UK) on a QuantStudio™ 7 Pro System (Applied Biosystems, Foster City, CA, USA), in accord with the validated protocol of the Italian National Reference Centre for Brucellosis [14]. Compared to culture, which is highly specific but has variable sensitivity over time, real-time PCR markedly increases the diagnostic sensitivity, reaching >90% on whole blood while maintaining nearly 100% specificity, whereas culture positivity declines after the acute phase of infection [25].

Serological tests targeted the humoral phase, with antibodies typically detectable from 5–8 weeks post-infection. SAT is very sensitive (70–99%) but less specific (40–83%). CFT and IFAT provide higher specificity (~97–100%) with moderate to high sensitivity (~87–97%). The SAT primarily detected IgM during early infection, while the CFT identified both IgM and IgG antibodies and IFAT the IgG [25,26].

All the serological test targeted antibodies specific to *B. canis*, using animal sera and antigen preparations homologous to rough *Brucella* strains, which is crucial because the use of smooth antigens can lead to false negative or inconsistent results [8].

## 3. Results

The study population consisted of diverse groups of dogs, varying in age, sex, and breed, representative of the regional population. Ages ranged from 2 months to 17 years (mean and median age of 3 years). The gender distribution was nearly equal, with 64 males, 62 females, and 2 individuals whose sex was unknown due to anonymization. Over 21 breeds were represented, with mixed breeds comprising 25.8% of the study population, followed by Golden Retrievers (18.5%) and Yorkshire Terriers (14.5%).

None of the tested dogs resulted positive for *B. canis* with any of the methods used. Based on these findings, the estimated maximum prevalence in the sampled population is calculated to be up to 3.5% (95% confidence interval) using binomial distribution. Group-specific prevalence estimates, calculated using the “rule of three” in the absence of positive detections, are reported in Table 2, showing that although no cases were identified, low-to-moderate prevalence cannot be excluded, particularly in groups with smaller sample sizes.

## 4. Discussion

Findings of the study represent sentinel information across selected risk groups, reflecting potential exposures related to the area (e.g., refugee-associated dog movements, as well as national and international transfers for hunting or breeding or link to previous outbreak). In this context, sentinel data is gathered from chosen groups with a higher likelihood of natural exposure to *B. canis*. Such data are not intended to estimate prevalence in the broader canine population, but rather to provide an early warning by signaling the potential introduction or circulation of the pathogen. Among the groups, the selected ones may represent valuable sentinels for early detection. However, considering the different strata analyzed, all having zero positives, the upper 95% confidence bounds ranged from 6.2% to 31.2%, indicating that low-to-moderate prevalence cannot be ruled out. Due to the limited sample size and the heterogeneity of the subpopulations considered, the present findings cannot be interpreted as a precise estimate of *B. canis* prevalence in the general canine population of Central Italy, but the absence of positive animals indicates that *B. canis* is unlikely to be endemic in the Umbria region. If the infection were circulating endemically, at least one positive case would have been expected in the surveyed population.

However, as undetected subclinical cases and sporadic introductions may pose a risk for pathogen establishment and spread [1,2], continuous risk-based surveillance remains essential in low-prevalence areas to prevent introduction and to enable early detection, in line with One Health strategies. Once introduced, *B. canis* may spread rapidly within canine populations, as demonstrated by an outbreak in a Dutch kennel following the introduction of a single infected dog [27]. Screening protocols should be strengthened and improved to ensure verification of *B. canis* status in dogs entering or moving within a region. Preparedness for implementing prophylactic measures in the event of a positive case is equally important. These strategies are integral to managing risks and safeguarding public and animal health [7,14].

Diagnostic limitations must also be considered in the present study, since false negatives may occur due to the latency of seroconversion or the absence of seroconversion in some infected animals, especially in chronic infections [1,2,7]. Nevertheless, the combination of different serological assays with complementary principles, together with the use of a rough antigen, was intended to increase diagnostic sensitivity and reduce the likelihood of missed cases, thus strengthening the robustness of the overall negative findings. Direct and indirect tests were applied in parallel and interpreted jointly. Their diagnostic windows are not perfectly overlapping, which is advantageous, as culture/PCR are most effective during bacteremia while serology detects post-seroconversion cases. This multimodal strategy improves overall sensitivity (~98–99%) at the cost of some specificity, an acceptable trade-off in a surveillance context [25].

Given that canine infection is the sole source of human exposure to *B. canis*, the absence of positive cases in dogs indicates a negligible zoonotic risk in the study area. This underscores the critical role of veterinary surveillance as a frontline measure for preventing human infections and supports the integration of animal data into One Health risk assessment frameworks [2,6].

Comparison with data from other Italian regions makes evident the regional differences existing in *B. canis* epidemiology. Apart from the recent outbreak in the Marche region, which involved 598 dogs with a seroprevalence of 46.1% [14], a study in the Veneto region (North-East Italy) reported a prevalence of 1.95% [28], highlighting regional variability within the country. Such regional differences may be influenced by various factors, including variations in dog population density, stray dog management practices, and the extent of international dog movements. This is particularly relevant in Italy, where health services are organized at the regional level, making region-specific data essential to support evidence-based decision-making and to adapt veterinary public health strategies to local epidemiological contexts.

This study adds valuable data to the limited body of knowledge on *B. canis* in Europe. The absence of positive cases aligns with findings from similar low-prevalence regions but contrasts with reports from high-risk areas, underscoring again the heterogeneity of epidemiological patterns. The regional focus, though limited, provides critical insights for targeted surveillance and reinforces the need for standardized diagnostic and reporting protocols across Europe.

Globally, the rise in *B. canis* cases highlights the urgency of establishing international guidelines on infection control and management in pets. Countries like the United Kingdom, which are implementing risk-based import controls and diagnostic screening for *B. canis*, provide valuable examples of how to mitigate the risk of introduction and spread [17]. Expanding these measures globally, especially in or near endemic regions, is essential. Some countries adopt extreme measures such as culling infected animals to prevent the spread of *B. canis*, reflecting the seriousness of its zoonotic potential and its impact on public and animal health [2,14,16,29,30].

In addition to regulatory measures, education and awareness for the general public, particularly targeting high-risk groups, are important. Veterinary professionals, kennel operators, and breeders must be equipped with the knowledge and tools to effectively manage infection risks. Public awareness campaigns targeting dog owners and adopters can also play a crucial role in reducing the likelihood of introducing infected animals [14,31,32]. In line with recent recommendations [2,7,32], practical measures should include mandatory testing for *B. canis* prior to national or international dog movements and before the introduction of new animals into breeding facilities, together with isolation of infected dogs and strict adherence to biosafety precautions when handling suspected cases, alongside improved awareness and training for both veterinarians and dog owners.

## 5. Conclusions

While no evidence of *B. canis* circulation was found in the surveyed canine population, the risk of sporadic introduction persists due to ongoing international movements and regional variability. Until robust international guidelines are established, *B. canis* will remain an underestimated threat to animal and public health. These findings underscore the importance of proactive, regionally tailored surveillance and comprehensive diagnostic protocols as key tools for early detection and control. Integrating veterinary data into One Health frameworks is essential for anticipating zoonotic threats and informing public health preparedness.

## Figures and Tables

**Table 1:** Population groups of dogs included in the study, their origin/ownership, diagnostic tests applied, and main epidemiological risk factors considered.

Group (Origin/Ownership)	n	Direct Tests (Culture + Real-Time PCR)	Serological Tests	Notes on the Risk of Population
Stray dogs in kennels (public)	47	Yes	Yes (SAT, CFT)	Public kennels: high turnover; uncontrolled background; potential exposure in public kennels
Breeding dogs (private)	22	Yes	Yes (SAT, CFT)	Risk of vertical/venereal transmission; frequent national and international movements
Blood donor dogs (private)	28	No (whole blood not available)	Yes on stored plasma (SAT, CFT)	Transfusion risk; the group includes many hunting dogs frequently traveling abroad
Refugee-associated dogs (private)	23	Yes	8 dogs with SAT, CFT; 15 dogs IFAT only (hemolyzed sera)	Unknown health status; origin from endemic areas; recent international relocation
Outbreak-linked dogs (private)	8	Yes	Yes (SAT, CFT)	Epidemiological link to a large outbreak; potential persistence of infection despite monitoring

**Table 2:** Group-specific prevalence estimates of *Brucella canis*, calculated using the “rule of three” (upper 95% confidence limit) in the absence of detected cases.

Group	n	Positives Detected	Maximum Estimated Prevalence (%)
Stray dogs in kennels	47	0	6.4% (≈3/47)
Breeding dogs	22	0	13.6% (≈3/22)
Blood donor dogs	28	0	10.7% (≈3/28)
Refugee-associated dogs	23	0	13.0% (≈3/23)
Outbreak-linked dogs	8	0	37.5% (≈3/8)
Total	128	0	2.3% (≈3/128)

## Data Availability

No datasets were generated or analyzed beyond those presented in the current study. All relevant data are included within the article.

## Abbreviations

The following abbreviations are used in this manuscript:

SAT	Serum Agglutination Test
CFT	Complement Fixation Test
IFAT	Indirect Immunofluorescence Antibody Test
PCR	Polymerase Chain Reaction
